# Carta ao editor sobre “Os desfechos da artroplastia anatômica total do ombro não foram afetados negativamente pela pandemia de COVID-19”

**DOI:** 10.1055/s-0045-1809511

**Published:** 2025-06-14

**Authors:** Hinpetch Daungsupawong, Viroj Wiwanitkit

**Affiliations:** 1 Consultor acadêmico particular, Phonhong, Vienciana, Laos.; 2 Saveetha Institute of Medical and Technical Sciences, Saveetha University, Chennai, Tamil Nadu, Índia.

Caro Editor,

O artigo “Os desfechos da artroplastia anatômica total do ombro não foram afetados negativamente pela pandemia de COVID-19” 1 é o tópico da discussão desta carta. Nesse estudo, a amplitude de movimento, a força e os resultados da fisioterapia foram comparados em pacientes submetidos à artroplastia total reversa do ombro (ATRO) em 2019 e 2020. Os achados demonstraram que a elevação para a frente, a rotação externa e a rotação interna melhoraram significativamente em ambos os grupos. Diferentemente dos pacientes de 2019, os de 2020 foram submetidos à fisioterapia por menos tempo e fizeram menos sessões. Em 2020, no entanto, no último acompanhamento, os pacientes relataram uma pontuação média baixa de dor.

O tamanho comparativamente pequeno da amostra desse estudo – 24 pacientes em 2019 e 27 em 2020–é uma de suas deficiências, que pode induzir viés e limitar a aplicação dos resultados. Além disso, o viés de memória e as imprecisões em relatos podem ser decorrentes da estratégia de coleta de dados, a saber, da dependência de medidas relatadas pelo paciente em contatos telefônicos feitos em 2022.

No futuro, seria benéfico realizar um ensaio clínico randomizado maior para aprender mais sobre as variações nos resultados em pacientes submetidos à ATRO em momentos diferentes. Além disso, pesquisas longitudinais poderiam ser realizadas para a avaliação dos impactos em longo prazo da duração e conclusão da fisioterapia nos resultados dos pacientes. Estudos futuros poderiam investigar as causas do declínio no número de sessões de fisioterapia concluídas e sua duração em 2020, bem como quaisquer efeitos que isso possa ter tido nos resultados funcionais e na satisfação geral do paciente. Considerando tudo, esse estudo oferece informações perspicazes sobre os desfechos de pacientes submetidos à ATRO e enfatiza a importância de se rastrear a duração e a conclusão da fisioterapia para a melhora dos desfechos.
